# 

**Published:** 1928

**Authors:** 


### Sir George Alfred Wills, Bart.

As we go to press we regret to hear of the death of SIR GEORGE ALFRED WILLS, Bart., D.C.L. Oxon., LL.D. Bristol, Pro-Chancellor, formerly Chairman of Council, and first Treasurer of the University, and one of its principal benefactors; and President of the Bristol General Hospital, to which also he made many generous gifts.

### The Medical Library of the University of Bristol,

WITH WHICH IS INCORPORATED

### The Library of the Bristol Medico-Chirurgical Society.

---

*The following donations have been received since the publication of the List in January,* 1928.

*June,* 1928.

| | |
|---|---|
| American Laryngological, Rhinological and Otological Society (1) .. .. .. | 1 volume. |
| American Otological Society (2) .. .. | 1 ,, |
| Miss A. M. Bullen (3) .. .. .. .. | 1 ,, |
| Michell Clarke Memorial Fund (4) .. .. | 1 ,, |
| Prof. Edgeworth (5) .. .. .. .. | 1 ,, |
| Faculdade de Medicina de Sao Paulo (6) .. | 1 ,, |
| Prof. Fawcett (7) .. .. .. .. | 2 volumes. |
| Prof. E. W. Hey Groves (8) .. .. .. | 19 ,, |
| Mrs. Harty (9) .. .. .. .. .. | 4 ,, |
| Medical Society of London (10) .. .. | 1 volume. |
| The Registrar (11) .. .. .. .. | 2 volumes. |
| E. E. Smith, Esq. (12) .. .. .. | 1 volume. |
| Dr. W. A. Smith (13) .. .. .. .. | 1 ,, |
| Surgeon-General of the U.S. Army (14) .. | 2 volumes. |
| Sir W. T. Thiselton-Dyer, K.C.M.G. (15) .. | 1 volume. |
| The Vice-Chancellor (16) .. .. .. | 1 ,, |
| C. H. Walker, Esq., F.R.C.S. (17) .. .. | 1 ,, |
| Sir Humphry Rolleston (18) .. .. .. | 1 ,, |

Unbound periodicals have also been received from Professor Hey Groves.

### THE ONE HUNDRED AND TWENTY-FOURTH LIST OF BOOKS.

The figures in round brackets refer to the figures after the names of the donors. The books to which no such figures are attached have either been bought from the Library Fund or received through the *Journal.*

| | | | |
|---|---|---|---|
| **Albee, F. H.** | *Bone-graft Surgery* | (8) | 1915 |
| **Balme, H.** | *Contribution of Christian Thought to Medicine* | | 1928 |
| **Barker, J. E.** | *Chronic Constipation* | | 1927 |
| **Barrett, C. R. B.** | *History of the Society of Apothecaries of London* | (12) | 1905 |
| **Barrington-Ward, L. E.** | *Abdominal Surgery of Children* | | 1928 |
| *Biological Therapy* | | (9) | 1925 |
| **Borchard, A., and Schmieden, V.** | *Die deutsche Chirurgie im Weltkrieg.* 2 vols., Ed. 2 | (8) | 1920 |
| **Braun, H.** | *Die Lagerung verletzter und erkrankter Gliedmassen* | (8) | 1928 |
| **Broderick, R. A.** | *Dental Bacteriology* | | 1926 |
| **Buxton, J. L. D.** | *Dental Pathology* | | 1927 |
| **Buxton, J. L. D.** | *Dental Surgery* | | 1927 |
| **Calot, F.** | *Indispensable Orthopedics* 2nd Ed. | (8) | 1921 |
| **Campbell, J. M.** | *The Protection of Motherhood* | (7) | 1927 |
| **Cancer Control** | *American Society for the Control of Cancer* | (8) | 1927 |
| **Clark, C. A.** | *Dental Radiography* | | 1926 |
| **Cotton, F. J.** | *Dislocations and Joint Fractures* 2nd Ed. | (8) | 1924 |
| **Crawford, A. M.** | *Materia Medica for Nurses* | | 1927 |
| **Crookshank, F. G.** | *Diagnosis and Spiritual Healing* | | 1927 |
| **Cumberbatch, E. P.** | *Diathermy* 2nd Ed. | | 1927 |
| **Cushing, H.** | *The Meningiomas* | | 1927 |
| **Dorland, W. A. N.** | *American Illustrated Medical Dictionary.* 14th Ed. | (17) | 1927 |
| **Fairbairn, J. S.** | *Obstetrics* | | 1926 |
| **Fitzwilliams, D. C. L.** | *The Tongue and its Diseases* | | 1927 |
| **Fremantle, F. E.** | *The Health of the Nation* | | 1927 |
| **Goodall, E. W., and Washburn.** | *Infectious Diseases* 3rd Ed. | | 1928 |
| **Herrick, C. J.** | *Fatalism or Freedom* | | 1927 |
| **Hornibrook, E. A.** | *Practical Birth Control* | | 1927 |
| *Infantile Paralysis in Vermont,* 1894–1922 | | (8) | 1924 |
| **Jamieson, J. D. H.** | *Operative Dentistry* | | 1927 |
| **Jeffrey, G. R.** | *Common Symptoms of an Unsound Mind* | | 1923 |

| | | | |
|---|---|---|---|
| **Kappis, M.** | *Organisation und Ordnungsgemasser betrieb des Operationssaals* | (8) | 1927 |
| **Kolodny, A.** | *Bone Sarcoma* | (8) | 1927 |
| **Leriche, R.** | *Treatment of Fractures.* 2 vols. | (8) | 1918 |
| **Llewellyn, Ll. J.** | *Aspects of Rheumatism and Gout* | | 1927 |
| **Lovett, R. W.** | *Lateral Curvature of the Spine* | (9) | 1910 |
| **McDonagh, J. E. R.** | *The Nature of Disease.* Part II. | | 1927 |
| **McKenzie, D.** | *Diseases of the Throat, Nose and Ear* | 2nd Ed. | 1927 |
| **Magnan, V.** | *Leçons cliniques sur les maladies mentales.* | 2nd Ed. (16) | 1893 |
| *Medical Department of the U.S. Army in the World War.* Vols. VII. and XIII. | | (14) | 1927–8 |
| **Mitchell, T. W.** | *Problems in Psychopathology* | | 1927 |
| **Mühsam, R.** | *Was kann und wann muss der praktische Arzt operieren* | (8) | 1928 |
| **Norman, H. J.** | *Mental Disorders* | | 1928 |
| *Nouveau Traité de médécine.* Vol. XVIII. | | (4) | 1928 |
| **O'Donovan, W. J.** | *Dermatological Neuroses* | | 1927 |
| **Payne, J. F.** | *English Medicine in the Anglo-Saxon Times* | (15) | 1904 |
| **Pearson, M. G., and Drummond, J.** | *Fractured Femurs* | (8) | 1919 |
| *Register of Dangerous Drugs* | | | —— |
| **Riviere, C.** | *Pneumothorax and the Surgical Treatment of Pulmonary Tuberculosis* | 2nd Ed. | 1927 |
| **Rolleston, H.** | *Idiosyncrasies* | (18) | 1927 |
| **Rose & Carless** | *Manual of Surgery* | 11th Ed. (8) | 1924 |
| **Ross, J. M.** | *Post-mortem Appearances* | 2nd Ed. | 1928 |
| **Schinz, H. R., et al. eds.** | *Lehrbuch der Röntgendiagnostik* | (8) | 1928 |
| **Senn, N.** | *Practical Surgery* | (8) | 1902 |
| **Smith, E. E.** | *Aluminum Compounds in Food* | (12) | 1928 |
| **Still, G. F.** | *Common Disorders and Diseases of Childhood.* | 5th Ed. | 1927 |
| **Syme, W. S.** | *Diseases of the Nose, Throat and Ear* | | 1927 |
| **Tandler, J.** | *Topographische Anatomie dringlichen Operationen.* | 2nd Ed. (8) | 1923 |
| **Taylor, E. H.** | *Operative Surgery* | (8) | 1914 |
| **Thomson, A. T.** | *A Conspectus of Pharmacopœias* | (3) | 1810 |
| **Tylecote, F. E., and Fletcher, G.** | *Diagnosis of Diseases of the Lungs* | | 1927 |
| **Waring, Sir H. J.** | *Surgical Treatment of Malignant Disease* | | 1928 |
| **Whiteford, C. H.** | *Surgical "Don'ts" and "Do's"* | 2nd Ed. | 1928 |
| **Wide, A.** | *Medical and Orthopedic Gymnastics* | 4th Ed. (9) | 1909 |
| **Wilson, P. D., and Cochrane, W. A.** | *Fractures and Dislocations* | (8) | 1925 |
| **Wilson, T. S.** | *Tonic Hardening of the Colon* | | 1927 |

TRANSACTIONS, REPORTS, JOURNALS, ETC.

| | | |
|---|---|---|
| *Annales da Faculdade de Medicina de Sao Paulo.* Vol. II. .. .. | (6) | 1927 |
| Annual Report of the Ministry of Health, 1926–27. VIII. .. .. .. | | 1927 |
| British Journal of Surgery. Index, 1913–1923 .. .. .. .. .. .. | (8) | 1923 |
| *Congreso (Segundo) Nacional de Medicina. Actas y Trabajos.* Tome 6 | (5) | 1927 |
| Dentists' Register, 1928 .. .. .. .. .. .. .. .. .. .. .. .. | | 1928 |
| Kelly's Directory of Bristol, 1928 .. .. .. .. .. .. .. .. .. | | 1928 |
| Medical Annual, 1928 .. .. .. .. .. .. .. .. .. .. .. .. .. | | 1928 |
| Medical and Dental Students' Register, 1927 .. .. .. .. .. .. | | 1928 |
| Medical Register, 1928 .. .. .. .. .. .. .. .. .. .. .. .. | | 1928 |
| Medical Society's Transactions. Vol. L. .. .. .. .. .. .. .. | (10) | 1927 |
| Minutes of the General Medical Council. Vol. 64 and Index, 1903–1927 | (11) | 1927 |
| Published Papers of the Middlesex Hospital School, 1926–27.. .. .. | (7) | 1927 |
| Surgical Clinics of North America, Vol. VIII. Nos. 1 and 2 .. .. .. | | 1928 |
| Transactions of the American Laryngological, Rhinological and Otological Society. Vol. 25 .. .. .. .. .. .. .. .. .. .. .. .. | (1) | 1927 |
| Transactions of the American Otological Society. Vol. 17. Part 3 .. | (2) | 1927 |
| Transactions of the American Ophthalmological Society. Vol. 25.. .. | | 1927 |
| Transactions of the Ophthalmological Society of the United Kingdom. Vol. 47 .. .. .. .. .. .. .. .. .. .. .. .. .. .. | | 1927 |

### Local Medical Notes.

Dr. G. Hadfield has been appointed Professor of Pathology in the University of London Royal Free Hospital School for Women.

Dr. M. G. Hughes has been appointed Assistant Medical Officer of Health to the City of Bristol.

Mr. Eric Watson-Williams has been appointed Honorary Surgeon, and Dr. J. Angell James Honorary Registrar, to the Ear, Nose and Throat Department of the Bristol Royal Infirmary.

Dr. A. L. Taylor, Lecturer on Pathology in the University of Leeds, has been appointed Pathologist to the Bristol

General Hospital and a Demonstrator of Pathology in the University of Bristol.

Dr. Newman Neild has been elected a Fellow of the Royal College of Physicians of London.

EXAMINATION RESULTS.

F.R.C.S. ENG.—Dr. J. Angell James.

**University of Bristol.**

M.B., CH.B. (*Part I.*).—*Second Examination.* *Pass :* R. N. O. Burns, R. G. C. G. Carlson, A. M. Davies, A. L. Eyre-Brook, H. F. M. Finzel, J. R. Gibbs, J. D. Hughes, J. J. Kempton, Frances N. Salisbury. *In Organic Chemistry* (*Completing Examination*) *:* C. J. N. Davis, C. N. Royal. *In Organic Chemistry only :* G. T. Bevir, W. R. Cambridge. *In Elementary Anatomy only :* J. Ridgway.

M.B., CH.B. (*Part II.*).—*Second Examination.* *Pass :* L. P. Ashton, K. P. Beaubrun, H. L. Cleave, J. L. S. Coulter, F. W. A. Fosbery, L. R. Jordan, J. A. Kersley, R. H. Moore, Elizabeth S. G. Owen, W. A. F. Taylor.

FINAL M.B., CH.B. (*Part I.*) (*including Forensic Medicine and Toxicology*)*:* Rowena M. Hickman, April Doreen James, Mabel F. Potter, N. L. Price (with Distinction in Materia Medica, Pharmacy, Pharmacology and Therapeutics). *In Forensic Medicine and Toxicology only :* Isabella J. Armstrong.

PART II. (*Completing Examination*)*:* T. H. Berrill (with Second Class Honours and Distinction in Special Pathology), B. J. Boulton, R. D. Jenkins (with Distinction in Public Health), Helen B. Murgoci (with Distinction in Special Pathology), T. B. Wansbrough (with Distinction in Obstetrics). *Group II.* (*Completing Examination*)*:* D. E. C. Andrew, A. J. McD. Grimston.

PRELIMINARY SCIENCE EXAMINATION FOR DENTAL STUDENTS.—*Pass :* G. N. Minifie, R. L. Royal, R. E. W. Smith.

B.D.S.—*Second Examination* (*Dental Anatomy only*). *Pass :* M. J. Ryan.

L.D.S.—*First Professional Examination.* *Pass :* J. D. Galloway. *In Dental Anatomy* (*Completing Examination*) *:* E. R. Carpenter, J. H. Edwards, N. Miller, I. W. Williams. *In Dental Anatomy only :* E. V. O'Hara, H. L. Saunders.

**Conjoint Board.**

M.R.C.S., L.R.C.P.—T. L. Cleave, G. Steinberg.

L.D.S., R.C.S.—*First Professional Examination. Part I. (Dental Mechanics):* J. H. White, W. Forsyth, H. Harwood.

L.D.S., R.C.S.—*First Professional Examination. Part II. (Dental Metallurgy):* G. E. Chadd, T. A. A. Eaves, W. F. G. Harvey, J. J. Hinton.

L.D.S., R.C.S.—*First Professional Examination. Part III. (b) (Dental Anatomy and Physiology):* D. R. Jones, W. R. R. Mewton, J. K. Noot, K. V. M. Taylor-Milton, A. H. V. Williams.

L.D.S., R.C.S.—*Final Examination. Part I.:* R. A. Phillips.

**Society of Apothecaries.**

L.S.A.—*Materia Medica and Pharmacy:* L. P. Gregory, G. Smith.

### Publications Received.

From Charles Griffin:

*Handbook of Medical Jurisprudence and Toxicology.* By W. A. Brend, M.A. (Camb.), M.D., B.Sc. (Lond.). 1928. Price 10s. 6d. net.

From Harrison & Sons Ltd., London:

*Surgical "Don'ts" and "Do's."* By C. H. Whiteford, M.R.C.S., L.R.C.P. Second (Enlarged) Edition. 1928. 4s. net.

From W. Hodge & Co., Edinburgh:

*On the Practice of "Terminal" Disinfection.* By Professor Carlos Chagas. 1928. 2s. 6d. net.

*Trauma and Compensation in Obstetric and Gynæcological Cases.* By D. M. Lindsay, M.D., F.R.F.P. & S.G. 1928. 7s. 6d. net.

From H. K. Lewis & Co.:

*Clinical Examination of the Lungs.* By E. M. Brockbank, M.D. (Vict.), F.R.C.P., and A. Ramsbottom, M.D. (Vict.), F.R.C.P. Second Edition. 1928. Price 5s. net.

*Acute Aplastic Anæmia.* By A. H. Smith. 1928. 6s. net.

*Clinical Examination of the Nervous System.* By G. H. Monrad-Krohn, M.D., F.R.C.P. Fourth Edition. 1928. 7s. 6d. net.

*The Pathology, Diagnosis and Treatment of Neoplasms Originating in the Walls of the Urinary Bladder.* By L. R. Fifield, F.R.C.S. (Eng.). 1928. Price 7s. 6d. net.

*The Injection Treatment of Varicose Veins.* By A. H. Douthwaite, M.D., M.R.C.P. (Lond.). Third Edition. 1928. Price 4s. net.

From E. & S. Livingstone:

*Mental Disorders.* By H. J. Norman, M.B., B.Ch. (Edin.), D.P.H. (Edin. and Glas.). 1928. Price 14s.

*Tuberculous Intoxications.* By Joseph Hollos, M.D. 1928. Price 10s. 6d. net.

*Dental Metallurgy.* [Outlines of Dental Science. Vol. XI.] By A. Johnstone Brown L.R.C.P., L.R.C.S. (Edin.), L.D.S. 1928. Price 7s. 6d. net.

*Dental Anatomy.* [Outlines of Dental Science. Vol. VI.] By D. Headridge, L.D.S. R.C.S. (Eng.) and S. K. Gibson, L.D.S. (Man.). 1928. 8s. 6d. net.

*Orthodontics.* By A. G. Wilson, L.D.S., R.C.S. 1928. Price 8s. 6d. net. [Outlines of Dental Science. Vol. XII.]

From Humphrey Milford, Oxford University Press:

*Diseases of the Larynx.* By H. Barwell, M.B. (Lond.), F.R.C.S. (Eng.). Third Edition. 1928. 12s. 6d. net.

*A Short History of Medicine.* By Charles Singer, M.A., M.D.,D.Litt. (Oxford). 1928. Price 7s. 6d. net.

*Crawford W. Long and the Discovery of Ether Anesthesia.* By Frances L. Taylor, M.D. With a Foreword by Francis R. Packard, M.D. 1928. Price 18s. net.

From John Murray:

*Chronic Constipation.* By J. Ellis Barker. With a Preface by Sir William Michigan. 1927. Price 7s. 6d. net.

*How to Start in General Practice.* By I. G. Briggs, M.R.C.S., L.R.C.P. 1928. 6s. net.

From Oliver & Boyd:

*Clinical Surgery.* By J. W. Dowden, M.B., F.R.C.S.E. 1928. 2s. net.

*Requisites and Methods in Surgery.* By C. W. Cathcart, C.B.E., M.A., M.B., C.M. (Edin.), F.R.C.S. (Eng. and Edin.), and J. N. J. Hartley, O.B.E., M.B. (Edin.), F.R.C.S. (Eng. and Edin.). 1928. 12s. 6d. net.

From Student Christian Movement:

*The Contribution of Christian Thought to the Science and Practice of Medicine.* By H. Balme, F.R.C.S. 1928. 6d. net.

From John Wright & Sons Ltd., Bristol:

*Medical Annual.* 1928. Price 20s. net.

*British Journal of Surgery.* April, 1928. Price 12s. 6d. net. (Annual Subscription 42s.).

*The Prescribing of Spectacles.* By A. S. Percival, M.A., M.B., B.C. (Cantab.). Third Edition. Price 15s. net.

### Bristol Medico-Chirurgical Journal.

Published Quarterly (royal 8vo reduced) under the auspices of the Bristol Medico-Chirurgical Society.

*An excellent means of advertising*

The only Medical Journal published in the West of England, and has an extensive circulation not only amongst the Medical men of Bristol, but also amongst those of the adjoining Counties and South Wales, and in exchange goes to all parts of the world.

Terms for Advertisements:

Whole Page, £2 2s., Half Page, £1 5s., Quarter Page, 15s. per issue.

PRICES FOR SPECIAL POSITIONS ON APPLICATION.

J. W. ARROWSMITH LTD.,

Advertising Agents,

11 Quay Street, BRISTOL.

